# Diagnostic ability of OCT parameters and retinal ganglion cells count in identification of glaucoma in myopic preperimetric eyes

**DOI:** 10.1186/s12886-020-01616-5

**Published:** 2020-09-22

**Authors:** Teresa Rolle, Beatrice Bonetti, Alberto Mazzucco, Laura Dallorto

**Affiliations:** Eye Clinic, Department of Surgical Sciences, University of Torino, Via Cherasco 23, Torino, Italy

**Keywords:** Ganglion cells, Glaucoma, Myopia, Optical coherence tomography

## Abstract

**Background:**

The aim of the study is to evaluate the diagnostic ability of OCT parameters and retinal ganglion cells (RGCs) count in identify glaucomatous disease in myopic preperimetric eyes.

**Methods:**

This was a cross-sectional observational study. The study group consisted of 154 eyes: 36 controls, 64 preperimetric (PPG), and 54 primary openangle glaucoma (POAG) eyes. Each group was divided into three subgroups based on axial length: emmetropic, myopic with axial length (AL) < 25 mm, and myopic with AL > 25 mm, to analyze the effect of myopia. The RGCs count was obtained using a model described later. As regard the influence of myopia on OCT parameters and RGC count, we performed Pearson’s correlation. The Area Under Receiver Operator Characteristics Curves (AUROC curves) evaluated which parameter had the best sensitivity and specificity in identifying glaucoma in myopic eyes.

**Results:**

In Pearson’s test, all Ganglion Cell Complex (GCC) thicknesses showed the weakest and less significant correlation with AL in all groups. All the AUROCs were statistically significant, and above 0.5. Inferior GCC and Global Loss Volume (GLV) showed the highest AUCs in all myopic group and the best diagnostic ability in distinguishing control from glaucomatous eyes. RGCcount showed good AUROC in all groups, with sensitivities of about 83% in myopic eyes, and specificity over 91% in all groups.

**Conclusions:**

GCC is the parameter less influenced by the AL, and the inferior GCC and the GLV have the best diagnostic performance. The RGCcount has good sensitivity and specificity, so it can be used as a complementary test in the diagnosis of glaucoma in myopic preperimetric eyes.

## Background

Myopia and glaucoma are two of the commonest causes of impaired vision in the world population. The number of people affected by myopia is estimated to be 5 billion by 2050 [1]; more than 100 million people currently suffer from blindness and irreversible visual impairment due to glaucoma [2, 3]. Myopic eyes have a higher risk of glaucoma [4]. The link between the two diseases seems to be the more easily deformable lamina cribrosa in myopic eyes. Myopic changes consist in longer axial lengths and greater vitreous chamber depths together with alterations in connective tissue which may increase susceptibility of optic disc to glaucomatous damage [5].

With the ophthalmoscopic evaluation alone, it can be difficult to distinguish glaucomatous damage from myopia for a number of reasons [6]. In myopia the use of structural or functional tests for the diagnosis of glaucoma is not fully reliable, for the presence of peculiar alterations such as posterior staphylomas or macular atrophy. Thus, it is absolutely necessary to pay close attention to distinguish the alterations of glaucoma from those of myopia to avoid overdiagnosis of glaucoma in myopic eyes [7]. Recently, empirical formulas derived from the combination of structural and functional measures to estimate the number of RGCs have been developed [8–11], and several studies state they are more accurate than the individual parameters they derive from for assessing the severity and progression of glaucoma [9–13].

## Methods

This cross-sectional observational study took place at the Glaucoma Center of the Eye Clinic, Department of Surgical Sciences, University of Turin, Italy. The methods conformed to the principles of the Declaration of Helsinki; we obtained the informed consent of all subjects and the approval of the Ethics Committee (University and Polyclinic San Giovanni Battista of Turin).

The inclusion criteria for admission of study subjects were: 18 ≤ age ≤ 80 years, best-corrected visual acuity (BCVA) ≥ 20/30, spherical equivalent between + 1.00D and − 1.00 D for emmetropic subjects, and between − 3.00D and − 7.00D for myopic subjects, and gonioscopic evidence of open angle. No subjects with previous ocular surgery, or with ocular, systemic or neurological pathologies causing perimetric defects were included. Patients with macular pathologic changes related to myopia were excluded from the study, as myopia is frequently associated with macular changes and degenerations that may affect ganglion cell count.

We performed a full eye examination, Fourier-Domain-OCT (FD-OCT RTVue-100 software version A4, 5, 0, 59; Optovue, Fremont, CA, USA) for peripapillary and macular imaging, and measurement of axial lenght with low-coherence interferometry system (Aladdin biometer, Topcon). Standard Automated Perimery was performed with program 24–2 of the Humphrey Field Analyzer (HFA; Carl Zeiss Meditec, Jena, Germany), using Swedish Interactive Threshold Algorithm (SITA) Standard strategy, with reliability criteria of fixation losses ≤20%, false positives and false negatives ≤33%. The study included three groups of subjects: preperimetric glaucomatous subjects (PPG), primary open-angle glaucoma (POAG) subjects, and a healthy group that was required to have negative family history of glaucoma and normal IOP (≤ 21 mmHg), visual field (VF) test and optic nerve head (ONH) appearance. PPG subjects had IOP > 21 mmHg, and changes in optic nerve (cup–disc ratio alteration/disc hemorrhages/rim notching/diffused or localized RNFL defects), but no defects on VF. POAG subjects had IOP > 21 mmHg, and glaucomatous alterations in optic disc and VF, as stated by Hodapp-Parrish-Anderson criteria for diagnosing glaucomatous damage [14]. For the evaluation of optic nerve head appearance, we used slit-lamp biomicroscopy of posterior segment with a 78-D lens. Each group was further divided into three subgroups based on axial length: emmetropic, myopic with AL < 25 mm and myopic with AL > 25 mm, in order to analyze the effect of mild and moderate myopia.

### FD-OCT RTVue-100

We used the Glaucoma Protocol of FD-OCT RTVue-100 to acquire RNFL thickness measurements. We detailed this protocol in a previous study (Rolle et al.) [15]

### Estimate of retinal and macular ganglion cell count

To estimate RGC number we used the model by Medeiros et al. [9–11, 16] based on the empirical formulas of Harwerth et al. [8].

We described this formula in detail in our previous article (Rolle et al.) [15].

### Statistical analysis

We used Microsoft Excel 2016 worksheets and the SPSS statistical program for Windows (version 19.0, SPSS Inc., Chicago, IL) for the collection, processing and statistical analysis of the results. The analysis of variance (ANOVA) and χ2 test was used to assess the comparability of the groups for continuous and dichotomic variables respectively.

**Fig. 1:**
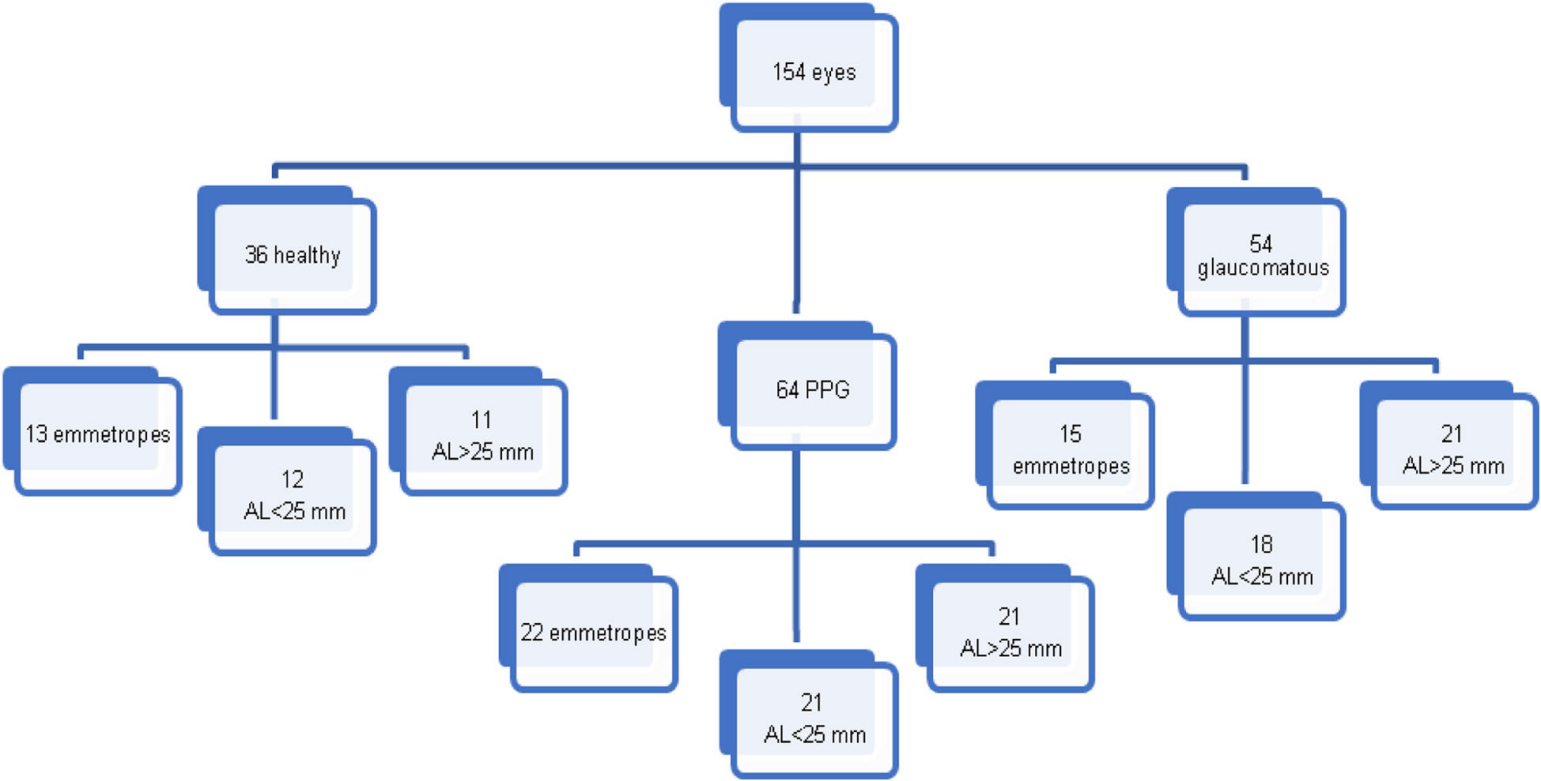
Graphical representation of the subdivision of the study sample into subgroups

For each of the three groups (controls, PPG and POAG) the Pearson’s linear correlation coefficient was calculated to evaluate how the measured parameters are influenced by the AL increasing. Then, each group was further subdivided into 3 subgroups (emmetropes, myopic eyes with AL < 25 mm and myopic eyes with AL > 25 mm) to analyze the effects of mild and moderate axial myopia by the Mann-Whitney U test (Fig. 1).

To investigate the ability of OCT parameters and RGCcount to diagnose glaucoma, we calculated areas under the receiver operating characteristic (AUROC). We compare the AUROCs between RGCcount and OCT parameters in all subgroups with different axial length (emmetropes, all myopic eyes, and myopes with AL < 25 mm and > 25 mm) using the method described by DeLong et al. [17]. For the calculation of AUROC and for all the comparisons between AUROCs we used a statistical software package (MedCalc v. 12.0; MedCalc Statistical software, Marakierke, Belgium).

For all statistical analysis, a *p* value < 0.05 was considered statistically significant.

## Results

The study group consisted of 154 eyes: 36 controls, 64 PPG, and 54 POAG eyes. Demographic characteristics of the study population are illustrated in Tables 1 and 2.

**Table 1:** Comparison of the Demographic and Clinical Characteristics of Control,PPG, and POAG Eyes

Parameters	Control	PPG	POAG	*P*-value	*P*-value for t test between groups		
					*Control* vs *PPG*	*Control* vs *POAG*	*PPG* vs *POAG*
Eyes (n)	36	64	54				
Age (y)	55.51 (7.72)	56.00 (7.28)	59.93 (7.28)	0.15*	0.82 ‡	0.06 ‡	0.18 ‡
Sex (M/F)	16/20	15/39	22/32	0.21*†*	0.102 ‡	0.73 ‡	0.16 ‡
Refraction (D)	−2.53 (2.45)	−2.49 (2.65)	−3.24 (2.74)	0.29*	0.93 ‡	0.21 ‡	0.13 ‡
Axial lenght (mm)	24.18 (1.16)	24.28 (2.19)	24.99 (2.75)	0.03*	0.78 ‡	0.04 ‡	0.03 ‡
MD (dB)	−0.48 (0.83)	−0.59 (1.64)	−6.50 (6.74)	< .001*	0.73 ‡	< .001 ‡	< .001 ‡
PSD (dB)	1.63 (0.37)	1.75 (0.96)	6.30 (3.63)	< .001*	0.42‡	< .001 ‡	< .001 ‡
RNFLavg (μ)	108.22 (10.90)	93.70 (9.95)	80.24 (13.2)	< .001*	< .001 ‡	< .001 ‡	< .001 ‡
GCCavg (μ)	97.11 (6.46)	85.52 (7.32)	75.19 (10.95)	< .001*	< .001 ‡	< .001 ‡	< .001 ‡
Estimated total RGC count (n)	1,140,520.81 (138,535.47)	958,579.68 (150,472.40)	616,463.06 (226,325.70)	< .001*	< .001‡	< .001 ‡	< .001 ‡

The parameters are expressed as mean and SD; *MD* Mean deviation of SAP, *PSD* Pattern standard deviation of SAP. *RNFL* Retinal Nerve Fiber Layer, *GCC* Ganglion Cell Complex, *RGC* Retinal Ganglion Cell

*P-value for ANOVA, significant for *p* < 0.05

†*P*-value for χ2 test, significant for *p* < 0.05

‡*P*-value for t test between groups, significant for *p* < α adjusted with Bonferroni correction (*p*-value with IC 95% / number of comparisons = 0.05/3 = 0.0167)

**Table 2:** Comparison of the Demographic and Clinical Characteristics of Emmetropic.Eyes. Myopes with AL < 25 mm and Myopes with AL > 25 mm

Parameters	Emmetropes	Myopes AL < 25 mm	Myopes AL > 25 mm	*P*-value	*P*-value for t test between groups		
					Emmetropes vs Myopes AL > 25 mm	Emmetropes vs Myopes AL < 25 mm	Myopes AL > 25 mm vs Myopes AL < 25 mm
Eyes (n)	50	51	53				
Age (y)	58.92 (8.67)	57.94 (10.76)	57.89 (11.36)	0.85*	0.62 ‡	0.61 ‡	0.98 ‡
Sex (M/F)	18/32	16/35	19/34	0.85*†*	0.62 ‡	0.84 ‡	0.63 ‡
Refraction (D)	0.64 (0.34)	−3.62 (0.43)	−5.24 (1.10)	< .001*	< .001 ‡	< .001 ‡	< .001 ‡
Axial lenght (mm)	22.82 (0.80)	24.41 (0.61)	26.23 (1.30)	< .001*	< .001 ‡	< .001 ‡	< .001 ‡
MD (dB)	−2.16 (4.43)	−2.18 (5.37)	−3.50 (5.30)	0.24*	0.96 ‡	0.14 ‡	0.16 ‡
PSD (dB)	2.77 (2.23)	3.39 (3.42)	3.76 (3.67)	0.27*	0.30 ‡	0.09 ‡	0.55 ‡
RNFLavg (μ)	96.43 (14.06)	95.23 (16.66)	86.02 (13.58)	0.002*	0.58 ‡	0.0005 ‡	0.008 ‡
GCCavg (μ)	87.63 (7.70)	85.12 (14.50)	81.59 (11.87)	0.04*	0.22 ‡	0.003 ‡	0.23 ‡
Estimated total RGC count (n)	941,639.43 (259,924.16)	918,347.12 (279,783.75)	786,566.29 (260,723.37)	0.007*	0.57 ‡	0.002 ‡	0.02 ‡

The parameters are expressed as mean and SD; *MD* Mean deviation of SAP*, PSD Pattern* standard deviation of SAP. *RNFL* Retinal Nerve Fiber Layer*, GCC* Ganglion Cell Complex*, RGC* Retinal Ganglion Cell

*P-values for ANOVA, significant for *p* < 0.05

*† P-values for χ*^*2*^ *test*, significant for *p* < 0.05

‡*P*-value for t test between groups, significant for *p* < α adjusted with Bonferroni correction (p-value with IC 95% / number of comparisons = 0.05/3 = 0.0167)

In Table 1 the ANOVA and χ^2^ test showed that the groups are comparable for age, sex, and refraction. T-test between groups shows that groups are comparable also for AL.

Glaucomatous eyes have significantly worse VF MD and PSD than control and PPG eyes (*P* < 0.001).

RNFLavg and RGC number reduce with the progression of glaucomatous damage, as demonstrated in previous studies [15, 18].

As regard the influence of myopia on OCT parameters and RGC count, we performed the Pearson’s correlation of axial length with RGC (Table 3). RNFL shows a significant negative moderate correlation with AL in all groups, while GCC has a weak positive correlation with AL not significant in control group, a weak negative correlation in PPG and POAG, statistically significant except for GCCavg and GCCsup in POAG group. FLV seems to be strongly correlated with AL (moderate significant positive correlation with AL in all groups), GLV has a significant positive moderate correlation with AL only in PPG group, while in control group the correlation is weak and not significant. Also RGCcount seems to be correlated with AL, because it has a moderate significant correlation in all groups.

**Table 3:** Pearson’s correlation between OCT parameters and RGC count and axial lenght

Parameters	Control	PPG	POAG
	R (p)	R (p)	R (p)
MD (dB)	−0.28 **(0.05)**	−0.07 (0.56)	−0.34 **(0.01)**
RNFLavg (μm)	−0.46 **(0.002)**	− 0.33 **(0.003)**	− 0.3 **(0.01)**
RNFLsup (μm)	− 0.31 **(0.03)**	− 0.28 **(0.01)**	− 0.3 **(0.03)**
RNFLinf (μm)	− 0.55 **(<.001)**	− 0.31 **(0.007)**	−0.3 **(0.01)**
GCCavg (μm)	0.07 (0.3)	−0.29 **(0.01)**	−0.15 (0.1)
GCCsup (μm)	0.1 (0.3)	−0.23 **(0.03)**	−0.04 (0.4)
GCCinf (μm)	0.045 (0.4)	−0.31 **(0.006)**	−0.24 **(0.04)**
FLV %	0.44 **(0.004)**	0.43 **(<.001)**	0.57 **(<.001)**
GLV %	0.22 (0.1)	0.48 **(<.001)**	0.26 **(0.03)**
RGCcount (n)	−0.58 **(<.001)**	−0.3 **(<.001)**	− 0.32 **(0.007)**

*P*-values significant for *p* < 0.05

To better evaluate the influence of myopia, we subdivided each group in three subgroups, basing on refractive error emmetropic eyes with defect between + 1.00 sf and − 1.00 sf, and myopic eyes with defect between − 3.00 sf and − 7.00 sf, and in these latter eyes axial length superior or inferior to 25 mm. This cut-off was chosen because it is two standard deviations from the reference average of the main normative databases [19].

In Tables 4, 5, 6 we resumed the results of Mann-Whitney U Test between PPG and control subgroups. In comparisons between control subgroups with different axial length (Table 6) we can merely evaluate the influence of myopia on OCT parameters. In all comparisons the MD is not statistically significant, a sign that there is no influence of any myopic damage on the functional aspect. In all the subgroups we can observe a difference between emmetropes and myopes for both macular and papillary OCT parameters, a sign of an influence of myopia on the structural aspect.

**Table 4:** RNFL and GCC measurement and RGCcount in PPG subgroups

Parameters	PPG emmetropic (*N* = 22)	PPG with AL < 25 mm (*N* = 21)	PPG with AL > 25 mm (*N* = 21)	PPG emmetropic vs PPG with AL < 25 mm	PPG emmetropic vs PPG with AL > 25 mm	PPG with AL < 25 mm vs PPG with AL > 25 mm
				*P*-values		
MD (dB)	− 0.98 (1.52)	0.18 (1.26)	−0.94 (1.89)	0.12	0.6	0.07
RNFL avg. (μm)	97.17 (10.82)	95.0 (8.05)	88.68 (9.07)	0.4	**0.009**	**0.02**
RNFL sup (μm)	98.01 (11.01)	93.52 (8.78)	90.30 (12.07)	0.11	**0.02**	0.21
RNFL inf (μm)	96.31 (11.84)	96.66 (10.66)	87.06 (11.22)	0.96	**0.02**	**0.008**
GCC avg. (μm)	88.20 (6.68)	86.73 (4.60)	81.52 (8.64)	0.28	**0.005**	**0.005**
GCC sup (μm)	88.99 (7.31)	87.86 (5.11)	82.82 (8.16)	0.29	**0.005**	**0.007**
GCC inf (μm)	87.40 (7.04)	85.66 (5.02)	80.22 (9.91)	0.3	**0.01**	**0.03**
FLV %	1.68 (2.22)	2.41 (2.74)	4.50 (4.67)	0.06	**0.01**	0.2
GLV %	9.59 (5.71)	12.29 (4.68)	18.07 (7.16)	0.08	**<.001**	**0.005**
RGC count (n)	1,002,394.31 (189,401.24)	986,664.15 (91,736.86)	884,594.19 (128,656.99)	0.96	**0.05**	**0.007**

The parameters are expressed as mean and SD. There is no difference between emmetropic and mild myopic PPG, while all the parameters analyzed are statistically significant in comparison between emmetropes and high myopic PPG, and between mild and high myopic PPG. *P*-values significant for *p* < 0.05

**Table 5:** RNFL and GCC measurement and RGCcount in PPG versus control eyes of comparable AL

Parameters	PPG emmetropic (*N* = 22)	PPG with AL < 25 mm (*N* = 21)	PPG with AL > 25 mm (*N* = 21)	Emmetropic Control (*N* = 13)	Control with AL < 25 mm (*N* = 12)	Control with AL > 25 mm (*N* = 11)	PPG emmetropic vs emmetropic control	PPG with AL < 25 mm vs control with AL < 25 mm	PPG with AL > 25 mm vs control with AL > 25 mm
							*P*- values		
MD (dB)	−0.98 (1.52)	−0.94 (1.89)	0.18 (1.26)	−0.12 (0.85)	− 0.64 (0.79)	− 0.73 (0.76)	0.06	0.05	0.92
RNFL avg. (μm)	97.17 (10.82)	88.68 (9.07)	95.09 (8.05)	109.76 (9.79)	114.54 (10.61)	100.15 (7.59)	**0.006**	**<.001**	**0.003**
RNFL sup (μm)	98.01 (11.01)	90.30 (12.07)	93.52 (8.78)	106.45 (9.32)	113.45 (13.76)	101.04 (10.04)	0.06	**<.001**	**0.01**
RNFL inf (μm)	96.31 (11.84)	87.06 (11.22)	96.66 (10.66)	113.08 (11.48)	115.62 (9.26)	99.30 (7.78)	**0.004**	**<.001**	**0.005**
GCC avg. (μm)	88.20 (6.68)	81.52 (8.64)	86.73 (4.60)	92.70 (4.37)	102.24 (6.68)	96.75 (4.12)	0.07	**<.001**	**<.001**
GCC sup (μm)	88.99 (7.31)	82.82 (8.16)	87.86 (5.11)	91.66 (4.96)	100.96 (7.20)	96.86 (5.37)	0.29	**<.001**	**<.001**
GCC inf (μm)	87.40 (7.04)	80.22 (9.91)	85.66 (5.02)	93.74 (4.05)	103.60 (6.56)	96.62 (3.73)	**0.02**	**<.001**	**<.001**
FLV %	1.68 (2.22)	4.50 (4.67)	2.41 (2.74)	0.20 (0.22)	0.34 (0.46)	1.00 (1.09)	**0.001**	**<.001**	**0.005**
GLV %	9.59 (5.71)	18.07 (7.16)	12.29 (4.68)	4.69 (2.76)	1.57 (1.50)	4.99 (2.21)	**0.006**	**<.001**	**<.001**
RGC count (n)	1,002,394.31 (189,401.24)	1,183,055.86 (125,256.44)	986,664.15 (91,736.86)	884,594.19 (128,656.99)	1,191,809.55 (129,243.34)	1,034,300.79 (109,779.01)	**0.006**	**<.001**	**0.005**

The parameters are expressed as mean and SD. RNFLavg and RGCs count are significant in all comparisons, while GCCavg is not significant in the comparison between PPG and emmetropes control. *P*-values significant for *p* < 0.05

**Table 6:** RNFL and GCC measurement and RGCcount in control eyes subgroups

Parameters	Emmetropic control (*N* = 13)	Control with AL < 25 mm (*N* = 12)	Control with AL > 25 mm (*N* = 11)	Emmetropic Control vs control with AL < 25 mm	Emmetropic Control vs control with AL > 25 mm	Control with AL < 25 mm vs control with AL > 25 mm
				*P*- values		
MD (dB)	−0.12 (0.85)	− 0.64 (0.79)	−0.73 (0.76)	0.11	0.12	0.83
RNFL avg. (μm)	109.76 (9.79)	114.54 (10.61)	100.15 (7.59)	0.31	**0.04**	**0.003**
RNFL sup (μm)	106.45 (9.32)	113.45 (13.76)	101.04 (10.04)	0.18	0.12	**0.003**
RNFL inf (μm)	113.08 (11.48)	115.62 (9.26)	99.30 (7.78)	0.81	**0.01**	**0.02**
GCC avg. (μm)	92.70 (4.37)	102.24 (6.68)	96.75 (4.12)	**<.001**	0.05	**0.03**
GCC sup (μm)	91.66 (4.96)	100.96 (7.20)	96.86 (5.37)	**0.004**	0.06	0.13
GCC inf (μm)	93.74 (4.05)	103.60 (6.56)	96.62 (3.73)	**<.001**	0.09	**0.005**
FLV %	0.20 (0.22)	0.34 (0.46)	1.00 (1.09)	0.60	**0.04**	**0.05**
GLV %	4.69 (2.76)	1.57 (1.50)	4.99 (2.21)	**0.005**	0.68	**<.001**
RGC count (n)	1,183,055.86 (125,256.44)	1,191,809.55 (129,243.34)	1,034,300.79 (109,779.01)	0.76	**0.01**	**0.007**

The parameters are expressed as mean and SD. *P*-values significant for *p* < 0.05

In Table 7 we compared emmetropic PPG eyes and all myopic PPG eyes with no distinguishing about axial length, and the difference is statistically significant for all parameters.

**Table 7:** RNFL and GCC measurement and RCGcount in subgroups with different AL, without distinction of disease stage

	Emmetropic PPG	Myopic^a^ PPG	Myopic Control^b^	Non-highly myopic^c^	Highly myopic^d^	Emmetropic PPG vs Myopic^a^ PPG	Myopic^b^ Control vs myopic^a^ PPG	Non-highly myopic^c^ vs highly myopic^d^
						*P*-values		
MD (dB)	−0.98 (1.52)	−0.38 (1.69)	−0.69 (0.76)	−2.12 (6.09)	−3.58 (5.16)	**0.04**	0.11	**0.04**
RNFL avg. (μm)	97.17 (10.82)	91.89 (9.07)	107.66 (11.68)	94.69 (13.23)	86.46 (13.87)	**0.03**	**<.001**	**0.002**
RNFL sup (μm)	98.01 (11.01)	91.91 (10.55)	107.51 (13.44)	95.05 (13.26)	88.15 (15.30)	**0.02**	**<.001**	0.06
RNFL inf (μm)	96.31 (11.84)	91.86 (11.85)	107.81 (11.82)	94.31 (14.57)	84.78 (14.88)	0.17	**<.001**	**<.001**
GCC avg. (μm)	88.20 (6.68)	84.12 (7.32)	99.61 (6.16)	84.77 (8.88)	81.61 (11.78)	**0.01**	**<.001**	**0.03**
GCC sup (μm)	88.99 (7.31)	85.34 (7.19)	99.00 (6.59)	86.15 (8.62)	83.43 (11.79)	**0.02**	**<.001**	**0.04**
GCC inf (μm)	87.40 (7.04)	82.94 (8.24)	100.26 (6.37)	83.41 (10.58)	79.78 (13.14)	**0.03**	**<.001**	0.06
FLV %	1.68 (2.22)	3.46 (3.93)	0.66 (0.87)	4.22 (3.74)	5.96 (5.39)	**0.002**	**<.001**	**0.009**
GLV %	9.59 (5.71)	15.18 (6.65)	3.21 (2.53)	15.19 (7.85)	18.39 (10.28)	**0.008**	**<.001**	0.06
RGC count (n)	1,002,394.31 (189,401.24)	935,629.17 (121,851.31)	1,116,479.27 (142,484.24)	910,940.59 (226,819.93)	784,036.59 (253,833.31)	**<.001**	**<.001**	**<.001**

The parameters are expressed as mean and SD. *P*-values significant for *p* < 0.05

^a^Myopic eyes with preperimetric glaucoma, without distinction for axial length

^b^Myopic control eyes with no distinction for axial length

^c^Control and PPG eyes with AL < 25 mm

^d^Control and PPG eyes with AL > 25 mm

**Fig. 2:**
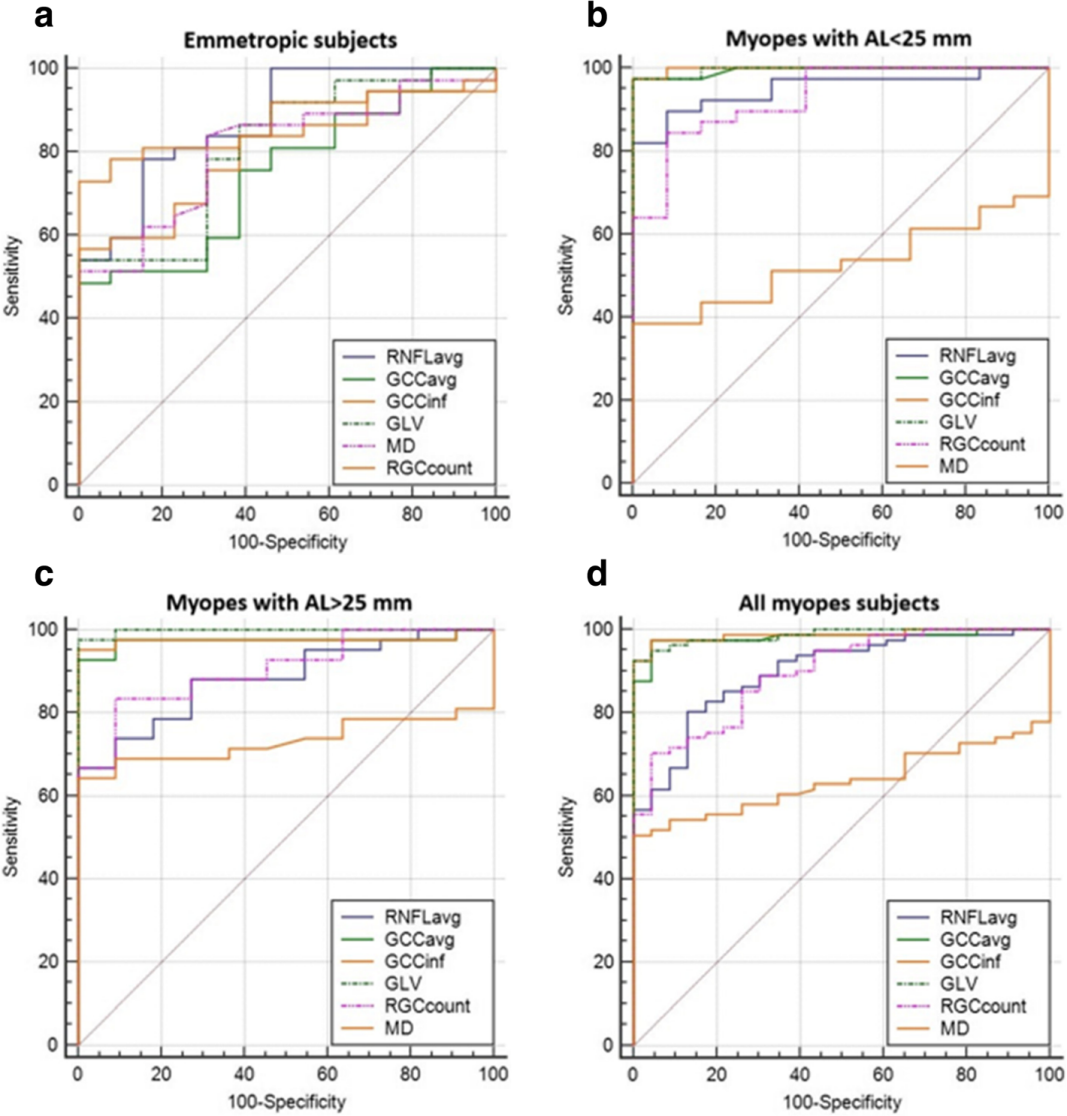
**a**-**d** AUROC of RNFLavg, GCCinf, GLV, RGCcount and MD in emmetropic eyes (**a**), myopes with AL<25 mm (**b**), myopes with AL>25mm (**c**) and all myopic eyes with no distinction in axial length (**d**)

The AUCs of MD, OCT parameters and RGCcount of emmetropes, myopes with AL < 25 mm, myopes with AL > 25 mm and all myopes subjects are summarized in Table 8 and Fig. 2;

**Table 8:** Comparison of AUROCs between RGCcount and OCT parameters, in all myopes groups

	Emmetropes			Myopes with AL < 25 mm			Myopes with AL > 25 mm			All myopes		
	AUC p *	Difference between areas (SE)	p	AUC p *	Difference between areas (SE)	p	AUC p *	Difference between areas (SE)	p	AUC p*	Difference between areas (SE)	p
RGC count (n)	0.873 *< .001*	/	/	0.929 *< .001*	/	/	0.905 *< .001*	/	/	0.895 *< .001*	/	
MD (dB)	0.807 *< .001*	0.0665 (0.0476)	0.16	0.528 *0.72*	0.402 (0.0792)	**<.001**	0.733 *0.0004*	0.172 (0.07)	**0.008**	0.636 *0.008*	0.25 (0.05)	**<.001**
RNFL avg. (μm)	0.877 *< .001*	0.00416 (0.0463)	0.93	0.951 *< .001*	0.0214 (0.354)	0.55	0.883 *< .001*	0.0216 (0.0497)	0.66	0.899 *< .001*	0.00322 (0.0285)	0.91
RNFL sup (μm)	0.798 *< .001*	0.0716 (0.0558)	0.2	0.912 *< .001*	0.0171 (0.0414)	0.7	0.859 *< .001*	0.0455 (0.0532)	0.39	0.87 *< .001*	0.0258 (0.0334)	0.44
RNFL inf (μm)	**0.88** ***< .001***	0.0107 (0.0496)	0.83	0.949 *< .001*	0.0192 (0.309)	0.53	0.872 *< .001*	0.0325 (0.0525)	0.54	0.885 *< .001*	0.0102 (0.032)	0.75
GCC avg. (μm)	0.748 *< .001*	0.125 (0.0704)	0.08	0.995 *< .001*	0.0652 (0.0375)	0.08	0.974 *< .001*	0.693 (0.0476)	0.42	0.981 *< .001*	0.0862 (0.0337)	**0.01**
GCC sup (μm)	0.673 *< .001*	0.197 (0.0821)	**0.02**	0.951 *< .001*	0.0214 (0.0451)	0.64	0.963 *< .001*	0.0584 (0.0489)	0.23	0.953 *< .001*	0.058 (0.0373)	0.12
GCC inf (μm)	0.801 *< .001*	0.0748 (0.0618)	0.23	**0.998** ***< .001***	0.0684 (0.0382)	0.73	**0.976** ***< .001***	0.07 (0.05)	0.13	**0.987** ***< .001***	0.0918 (0.0329)	**0.005**
FLV %	**0.897** ***< .001***	0.0278 (0.0731)	0.7	0.949 *< .001*	0.0192 (0.0502)	0.7	0.9 *< .001*	0.00433 (0.0555)	0.94	0.917 *< .001*	0.0215 (0.0386)	0.58
GLV %	0.818 *< .001*	0.0603 (0.0705)	0.39	**0.996** ***< .001***	0.0662 (0.0369)	0.07	**0.998** ***< .001***	0.09 (0.04)	0.03	**0.987** ***< .001***	0.0913 (0.0312)	0.004

*Area under receiver operating characteristic curve values and p-values describing glaucomatous diagnostic capabilities of RGCcount and OCT parameters in emmetropic eyes, all myopic eyes, myopes with AL < 25 mm eyes and myopes with AL > 25 mm eyes

*P*-values significant for *p* < 0.05

All the AUROCs are statistically significant, and above 0.5. Inferior GCC and Global Loss Volume (GLV) show the highest AUCs in all myopic groups and the best diagnostic ability in distinguishing control from glaucomatous eyes. Both GCCinf and GLV have sensibility > 95% in all groups, except GLV sensitivity in all myopes’ group (92.59%).

RNFLavg and MD are used in the formula of RGCcount of Medeiros et al. [9] to calculate the RGC number. We compared the diagnostic performance of both parameters with that of RGCcount (see Table 8), using the method of De Long [17], to assess whether the retinal ganglion cell count adds more diagnostic information than the respective parameters from which it is derived. RGCcount performs significantly better than MD in all groups, and almost the same than RNFLavg with no statistical significant differences.

## Discussion

Patients with high myopia has a sixfold increased odds to develop glaucomatous disease [20], and in this case the early diagnosis is mandatory and needs tests with high sensitivity and specificity [21].

The evaluation of peripapillary RNFL is used in common clinical practice to detect the presence of glaucomatous damage [22], but in high myopia its interpretation is made difficult by the frequent presence of optic nerve tilt. Shin et al. [23] showed that optic disc tilt reduce RNFL diagnostic ability in detecting glaucoma, while it doesn’t influence ganglion cell-inner plexiform layer (GCIPL) thickness, which is more reliable in the evaluation of glaucoma in high myopia.

Also Chen et al. [24] argue that evaluation of glaucoma with nonhighly myopic database could lead to misdiagnosis, and that GCC thickness determined by a myopic database should be used.

In our study RNFL and RGCs count are correlated to the increase in axial length with a moderate significant negative correlation, while GCC thicknesses seem to be the least correlated to axial length increase because they have a weak correlation in all groups, not significant in control group where there is no confounding factor of glaucomatous disease. (Table 3). This is this is in agreement with the results of many studies: Shoji et al. [25] have shown that GCC parameters are not significantly affected by high myopia, while RNFL measurements have a decreased ability to detect glaucoma in myopic subjects. Wang [26] and Scuderi [27] have established that macular GCC thickness has higher diagnostic power than peripapillary RNFL thickness in high myopia.

Considering the results of the comparisons among subgroups (Tables 4, 5, 6 and 7) there are statistically significant results for almost all the parameters in comparison between control group and PPG, both among the myopes and emmetropes. So we could support, according to the studies of Tan et al. [28] and Kim et al. [29], that the RNFL and GCC parameters are complementary in the evaluation of glaucomatous damage also in myopic eyes.

As regards diagnostic ability of OCT parameters, in our study all parameters has an AUROC > 0.5 (Table 8), and all curves are statistical significant, with high values of sensibility and specificity. While RNFLinf and FLV showed the best AUROCs in emmetropes, GCCinf and GLV showed the best AUROCs in all myopic group. This is in agreement with many studies that demonstrated that GCC thickness have a glaucoma detection ability as effective as that of RNFL parameters [25, 28–33].

However we must take into account some limitations of GCC in the evaluation of glaucoma in myopic eyes. In the eyes in which the head of the optic nerve is deformed and therefore difficult to evaluate, we may assume that the macular region is less distorted, but this is not always true. The studies by Kim et al. [34, 35] have suggested that the outline of the entire posterior pole determines the possible configuration of the optic nerve head. So, the presence of irregularities in macular region could invalidate the evaluation of GCC in myopia. Another bias is due to the high axial length which causes a false positive GCC thinning [36]. This is because a greater axial length determines a streching of the globe with an increase in the distance between the optic nerve and the macula and consequent false thinning of macular region [37, 38]. Furthermore, the presence of macular degeneration can cause GCC thinning (for retinal atrophy) or thickening (for intraretinal fluid due to myopic CNV or to macular retinoschisis) that are independent from glaucoma [39].

To our knowledge, no previous studies have reported on use of RGCs count in identify glaucoma in myopic eyes and its diagnostic ability. There are many studies about RGCcount in non-myopic eyes demonstrating that a combined measure of structural and functional parameters performs better than the single OCT and perimetry parameters [13, 16]. We wanted to evaluate if the diagnostic ability of the RGC was superior to those of the single parameters used in its calculation formula (MD and RNFLavg) also in myopic groups: both in mild and high myopes, AUROCs of RGCcount are significantly better than those of MD, and approximately similar to those of RNFLavg without statistically significant differences (Table 8). In all groups, both myopes and emmetropes, RGCcount shows very good AUROCs (between 0.873 in emmetropes and 0.929 in mild myopes), with sensitivity > 70% and specificity > 90%.

Based on the results of our study, RGCs count seems to be complementary to OCT parameters in the detection of glaucomatous damage in the myopia, also if GCC parameters show better diagnostic ability. Since the glaucomatous damage in the myopia is more early to be detected at macular level, it could be useful to evaluate the number of macular ganglion cells, as already done by Rolle et al. [15], also in myopic subjects. This could be analysed in a further study.

The limitation of this study is that, despite of a good number of the total sample, the subdivision in different subgroups makes the number of each subgroup reduced. However, this is also found in other studies [40–42]. To validate the results obtained it would be indicated to use an even larger cohort of investigation. In addition, the cross-sectional design of the study is a weak point that limited longitudinal analysis, in fact we are carrying out the creation of a prospective cohort to evaluate the progression of VF in the preperimetric group.

Another limitation is related to the fact that the sample of myopic eyes does not perfectly correspond to what we find in clinical practice, because it does not include all the eyes with perimetric alterations (enlarged blind spot, general reduction of sensitivity and superotemporal peripheral defects), which also are very frequent in myopic eyes.

A strength is represented by the use of preperimetric eyes, since comparing only eyes diagnosed with both functionally and structurally established glaucoma with a control group would lead to overestimate the performance of the test, as reported by Medeiros et al. [43].

## Conclusions

In conclusion, in the OCT analysis of myopic eyes RNFL is the parameter most influenced by the axial length, while the GCC, in particular the inferior, and the GLV are the two OCT parameters with better diagnostic performance. The RGCcount appears to have good sensitivity and specificity, but not higher than the OCT parameters, so it can be used as a complementary test in the diagnosis of glaucoma in myopic eyes.

Identifying the presence of glaucoma in a myopic eye is one of the current diagnostic challenges in ophthalmology, and we must interpret all the instrumental data considering the influence of myopia. Current OCTs analyze the thicknesses of retinal nerve fibers and ganglion cells using a normative database that includes emmetropic subjects. Our study agrees with other works affirming that the OCT parameters are affected, although to varying degrees, by the axial length. Therefore to increase the reliability of the OCT in the diagnosis it would be appropriate to insert myopic eyes in the normative databases of the instruments and develop algorithms that take into account the axial length to analyze the thicknesses detected with OCT.

## Data Availability

The data have not been placed in any online data storage. The datasets used and analysed during the current study are available from the corresponding author on reasonable request.

## Abbreviations

AL: Axial Length
BCVA: Best Corrected Visual Acuity
FD: Fourier-Domain
FLV: Focal Loss Volume
GCC: Ganglion Cell Complex
GLV: Global Loss Volume
IOP: Intra-Ocular Pressure
MD: Mean Deviation
OCT: Optical Coherence Tomography
ONH: Optic Nerve Head
POAG: Primary Open Angle Glaucoma
PPG: Preperimetric Glaucoma
PSD: Pattern Standard Deviation
RGC: Retinal Ganglion Cell
RNFL: Retinal Nerve Fiber Layer
VF: Visual Field
